# CO_2_ as an engine for neurofluid flow: Exploring the coupling between vascular reactivity, brain clearance, and changes in tissue properties

**DOI:** 10.1002/nbm.5126

**Published:** 2024-02-25

**Authors:** Elisabeth C. van der Voort, Yunjie Tong, Eva E. van Grinsven, Jaco J. M. Zwanenburg, Marielle E. P. Philippens, Alex A. Bhogal

**Affiliations:** 1 Center for Image Sciences, UMC Utrecht, Utrecht, The Netherlands; 2 Purdue University, West Lafayette, Indiana, USA; 3 Radiotherapy, UMC Utrecht, Utrecht, The Netherlands

**Keywords:** BOLD MRI, cerebral blood volume, cerebrospinal fluid, cerebrovascular reactivity, peritumoral edema

## Abstract

The brain relies on an effective clearance mechanism to remove metabolic waste products for the maintenance of homeostasis. Recent studies have focused on elucidating the forces that drive the motion of cerebrospinal fluid (CSF), responsible for removal of these waste products. We demonstrate that vascular responses evoked using controlled manipulations of partial pressure of carbon dioxide (PaCO_2_) levels, serve as an endogenous driver of CSF clearance from the brain. To demonstrate this, we retrospectively surveyed our database, which consists of brain metastases patients from whom blood oxygen level-dependent (BOLD) images were acquired during targeted hypercapnic and hyperoxic respiratory challenges. We observed a correlation between CSF inflow signal around the fourth ventricle and CO_2_-induced changes in cerebral blood volume. By contrast, no inflow signal was observed in response to the nonvasoactive hyperoxic stimulus, validating our measurements. Moreover, our results establish a link between the rate of the hemodynamic response (to elevated PaCO_2_) and peritumoral edema load, which we suspect may affect CSF flow, consequently having implications for brain clearance. Our expanded perspective on the factors involved in neurofluid flow underscores the importance of considering both cerebrovascular responses, as well as the brain mechanical properties, when evaluating CSF dynamics in the context of disease processes.

## 1 | INTRODUCTION

The brain’s high metabolic rate necessitates an efficient clearance mechanism to eliminate byproducts and maintain long-term homeostasis. However, because of the absence of a conventional lymphatic system in the brain, the exact pathways for waste clearance remain a subject of debate.^1^ Consequently, research interest is growing, with a specific focus on understanding the forces that facilitate waste removal from the brain. Both cerebrospinal fluid (CSF) and interstitial fluid (ISF) are recognized as major carriers of waste products.^2^ Various mechanisms have been proposed to generate the necessary motive force for stimulating the circulation and eventual clearance of these neurofluids. For instance, studies have demonstrated that arterial dynamics, related to heartbeat and vasomotion, significantly impact CSF flow.^3–6^ In awake mice, increasing the amplitude of vasomotion through visually evoked vascular responses has been found to enhance clearance rates.^6^ Recent research in humans has shown similar findings, linking neurovascular coupling to macroscopic CSF flow.^7,8^ These collective findings underscore the pivotal role of cerebrovascular responses, specifically those leading to changes in cerebral blood volume (CBV), in driving brain waste clearance.

In addition to vascular contributions, studies have established a correlation between CSF flow and respiration. Proposed mechanisms for this correlation encompass intrathoracic and abdominal pressure changes,^9–11^ as well as fluctuations in partial pressure of carbon dioxide (PaCO_2_) levels^12,13^ during the respiratory cycle. Notably, carbon dioxide (CO_2_) serves as a potent vasoactive agent capable of modulating vascular tone, leading to changes in cerebrovascular reactivity (CVR) response, CBV, and cerebral blood flow (CBF)—in this way, respiration is also linked to cerebrovascular responses. The Monro–Kellie doctrine emphasizes maintaining a constant intracranial volume, thus changes in CBV are balanced by concurrent alterations in intracranial CSF volume.^14^ The link between vascular responses and CSF flow indicates that alterations in vascular compliance or impaired CVR response caused by disease could conceivably reduce the capacity for waste clearance. Nevertheless, we propose that the inverse may also hold true: changes in the ability for CSF (or ISF) to move freely throughout the brain may result in mechanical resistances that affect vascular responsiveness.

The primary objective of this study was 2-fold: first, to establish a direct link between CVR and CSF dynamics through controlled manipulations of arterial blood gases; and second, to investigate whether changes in the mechanical properties of the brain, potentially influencing neurofluid flow, impact CVR. To achieve this, we conducted a retrospective analysis on functional MRI (fMRI) blood oxygen level-dependent (BOLD) data acquired during precise manipulations of oxygen (PaO_2_) and PaCO_2_ by means of targeted respiratory challenges. The inclusion of the O_2_ component served as a means of validation, given its limited vasoactive effect during brief increases in arterial O_2_.^15^ Our study cohort comprised patients with brain metastases undergoing radiotherapy treatment who displayed varying degrees of cerebral edema. The presence of edema can lead to local changes in hydrostatic pressure and potentially cause widespread alterations in the mechanical properties of the brain. This unique cohort allowed us to explore the relationship between edema load and its effect on vascular response dynamics, and, by association, CSF flow dynamics. As a result, we offer a novel physiological perspective that connects brain clearance properties with vascular markers that have previously been associated with vascular impairment.

## 2 | METHODS

### 2.1 | Data acquisition

Data were retrospectively selected from the ongoing Assessing and Predicting Radiation Influence on Cognitive Outcome using the cerebrovascular stress Test (APRICOT) study.^16^ The cohort consists of adult patients (age ≥ 18 years) with either radiographic and/or histologic proof of brain metastases that were referred to the radiotherapy department of the UMC Utrecht (UMCU) for radiotherapy treatment. Patients were scanned prior to and approximately 3 months after radiotherapy. The study was performed in accordance with the Declaration of Helsinki and the UMCU institutional ethical review approved the study. Written informed consent was obtained from all participants prior to participation.

**FIGURE 1:**
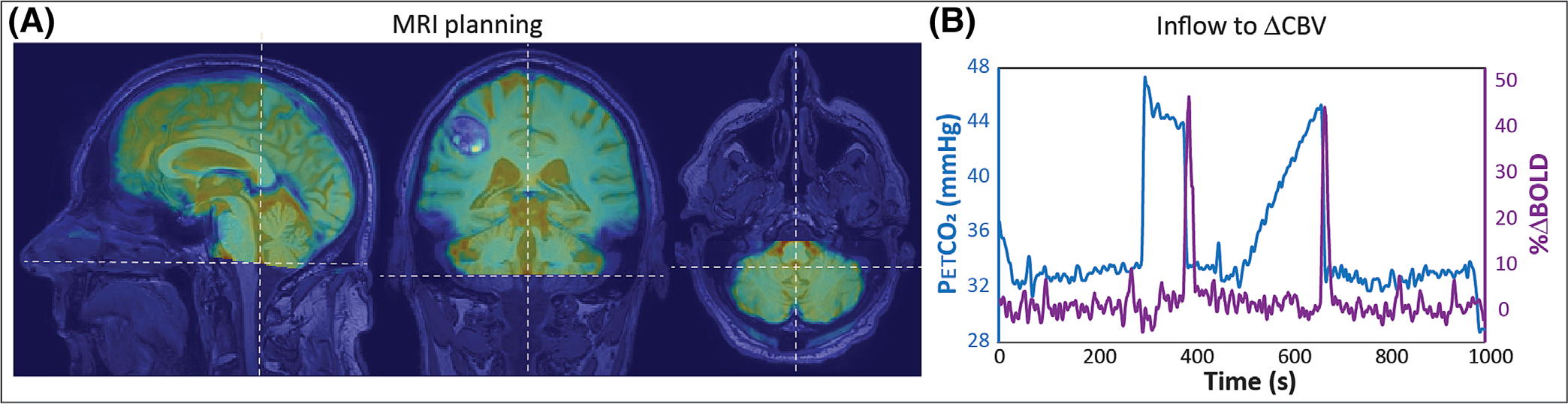
Single subject results: (A) Blood oxygen level-dependent (BOLD) data overlaid on T1-weighted image showing the planning of lower slices just below the fourth ventricle. (B) End-tidal CO_2_ trace (blue) and cerebrospinal fluid inflow signal (purple) as measured in the bottom slices. CBV, cerebral blood volume.

Patients were scanned on a 3-T scanner (Philips Healthcare, Best, The Netherlands) using a 32-channel receive coil. The MRI protocol consisted of a T2*-weighted gradient echo EPI sequence with the following parameters: repetition time (TR) = 1050 ms, echo time (TE) = 30 ms, flip angle 65, voxel size = 2.3 × 2.3 × 2.5 mm^3^, acquisition matrix = 96 × 96, 51 slices, 1000 volumes, and multiband factor = 3. Planning was aimed to obtain full brain coverage (Figure 1A). MRI data were acquired during the manipulation of arterial blood gases using a computer-controlled gas delivery system (RespirAct, Thornhill Medical, Toronto, Canada). Using prospective targeting, measured end-tidal pressure of CO_2_ (PetCO_2_) has been shown to be in agreement with PaCO_2_, and so, for the remainder of this work, these terms will be used interchangeably.^17^ The respiratory paradigm consisted of a 90-s hypercapnic block (+10 mmHg from baseline PetCO_2_), a progressively increasing 120-s hypercapnic ramp (max. + 12 mmHg from baseline), and finally a 180-s hyperoxic block (target: 680 mmHg). Each epoch was interleaved with a 120-s baseline period, during which arterial (C)O_2_ levels were maintained at individual predetermined baseline values. In addition to the fMRI scan, a 3D spoiled gradient echo sequence (TR = 8 ms, TE = 3.25 ms, flip angle 10°, isotropic resolution of 1 mm, acquisition matrix = 240 × 240 × 180) and a 3D T2-weighted fluid-attenuated inversion recovery (FLAIR) sequence (TR = 4800 ms, TE = 240 ms, TI = 1650 ms, flip angle 90°, isotropic resolution of 1 mm, acquisition matrix = 256 × 256 × 182) were acquired for tissue and edema segmentation, respectively.

### 2.2 | Data processing

The fMRI data were preprocessed using FSL.^18^ This consisted of motion correction (MCFLIRT), distortion correction (TOPUP), brain extraction (BET), which also was used to generate the whole-brain mask, and spatial normalization to the T1-weighted anatomical image (FLIRT). The T1-weighted images were then segmented (FAST) and the resulting gray matter (GM), white matter (WM), and CSF segmentations were transformed to co-register with the fMRI data. Edema segmentation based on the T2FLAIR image was performed using the lesion growth algorithm from the Lesion Segmentation Tool (LST) for SPM.^19^ When necessary, to eliminate both false positives and negatives, the resulting edema masks were manually corrected to include hyperintense regions that were missed by the automated tool. Finally, edema masks were co-registered with the fMRI data.

The average GM BOLD fMRI signal was calculated for each patient. To focus on presumed healthy tissue, and therefore the most robust vascular responses, tissues containing edema were excluded based on the T2FLAIR edema segmentations. To add a margin for error, edema masks were dilated by one voxel. The mean regional GM signal was calculated and then temporally de-noised using a wavelet-based approach implemented in the “denoiseData.m” function of the seeVR toolbox^20^ (wavelet parameters: family = sym4, level = 2, de-noising method: universal threshold, level-independent hard threshold). The temporal derivative was then calculated as a surrogate for CBV change.^21^ Respiratory traces were interpolated to the TR of the fMRI scans and a bulk alignment between the average whole-brain BOLD signal and resampled PetCO_2_ trace was performed.^20^

### 2.3 | Data analysis

#### 2.3.1 | Linking CSF flow and vascular response

All preradiotherapy and postradiotherapy datasets were screened for the presence of CSF inflow signal. Frequently, radiotherapy treatment can lead to significant widespread physiological changes associated with tumor regression. Moreover, in cases where treatment response was not positive, tumor progression may also manifest in widespread changes (e.g., increases in peritumoral edema). For these reasons, we chose to treat preradiotherapy and postradiotherapy datasets as individual subjects for our primary analysis. Voxels containing CSF near the fourth ventricle were manually selected by isolating the signals with the highest magnitude signal contrast in one of the two bottom slices of the imaging stack, as reported in previous studies.^8,21,22^ If both slices contained voxels with CSF inflow, the average of both traces was calculated. If no visible signal peak was found following the negative changes in PetCO_2_, data were excluded from this analysis. The resulting signals were de-noised as outlined above. CSF inflow peaks were analyzed with respect to the temporal derivative of both the GM BOLD fMRI signal and the CO_2_ trace performing a shifted cross-correlation that also provided a lag value for each comparison.

#### 2.3.2 | Relationship between vascular response and edema load

**FIGURE 2:**
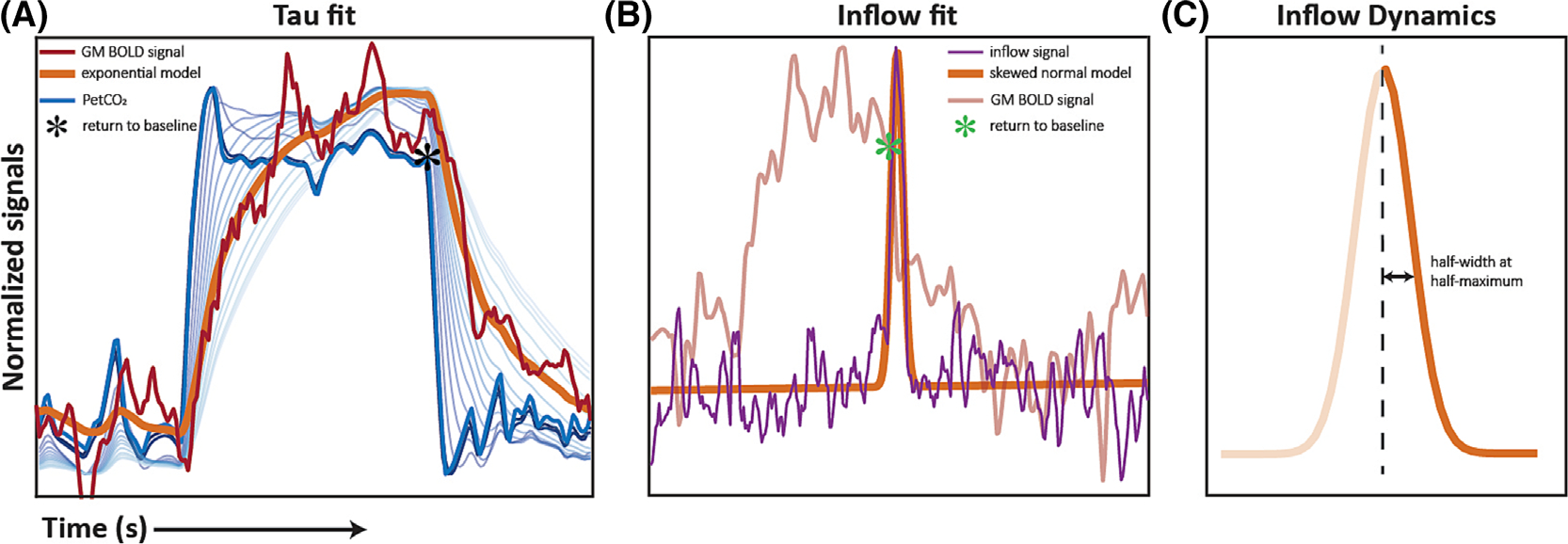
Schematic illustration of tau and inflow peak fit: (A) Gray matter blood oxygen level-dependent (GM BOLD) signal (red) and corresponding PetCO_2_ trace (blue) of one subject. The PetCO_2_ trace, after convolution with an exponential function with increasing tau values, is shown in the background. A least squares fit is performed between the convolved PetCO_2_ trace and the BOLD signal time series to identify tau (orange). (B) The cerebrospinal fluid (CSF) inflow signal (purple) of the same subject with the estimated fit (orange) based on a skewed normal probability density function. In both plots, the asterisk identifies the start of the transition to baseline, where inflow is also expected to begin. (C) The estimated CSF inflow fit showing the half width at half maximum.

The vascular response dynamics were evaluated under the assumption that they provided a surrogate marker of CSF flow dynamics. This was done for all datasets, both preradiotherapy and postradiotherapy, assuming that the brain composition and, consequently, the vascular dynamics, would be notably different between both time points. For this, the PetCO_2_ trace for the hypercapnic block was isolated within the protocol range of imaging volume 250–450 (262.5–472.5 s). A least squares fit was then performed between the individual GM time series and the associated PetCO_2_ trace after convolution with an exponential hemodynamic response function, as described by Poublanc et al.^23^ (Figure 2A). This was done using the “fitTau1D.m” function of the seeVR toolbox. The time constant of the exponential curve, defined as “tau”, was taken to represent the CVR response dynamics. The hypercapnic ramp portion of the protocol was not included in this analysis because the gradual increases in arterial CO_2_ do not evoke the sudden changes in CBV needed to probe the response dynamics.

#### 2.3.3 | Relationship between vascular dynamics and CSF dynamics

To evaluate CSF inflow dynamics, the half-width of the CSF inflow peak was estimated in all datasets. This was done by performing a multiple least squares fit of the inflow signal with a normal probability density function that included a skewness factor and a linear function to account for baseline signal effects (Figure 2B). The Matlab code used to perform fitting is provided in Data S1. The data range used for the fit began at the end of the hypercapnic block (imaging volume 250) and ended at the midpoint between the end of the hypercapnic block and the peak of the hypercapnic ramp (imaging volume 525). This endpoint was chosen to include the entire tail of the CSF inflow peak in cases where the response time was long. The half width at half maximum (HWHM) for the right side of the normal probability density function was then taken as a proxy for the width of the CSF inflow peak (Figure 2C). To compare the CSF dynamics with the vascular dynamics (tau), only the width of the CSF peak associated with the hypercapnic block was considered.

The edema percentage was calculated in all datasets using the number of voxels contained within the edema mask relative to the corresponding whole-brain mask. Linear regression was performed to express tau as a function of both CSF inflow duration and edema load in the brain.

## 3 | RESULTS

Our retrospective survey of the patient database identified 30 potential subjects, of whom 18 had undergone a second scan session. A total of eight subjects were excluded because of missing or incorrect data (e.g., failed hypercapnic challenges). Of the remaining 22 subjects (15 males, 7 females, age 65.7 ± 8.2 years), 12 had repeated measurements, resulting in a total of 34 potentially useful datasets. Information regarding age, sex, and tumor etiology of all 22 subjects can be found in Data S1. In total, 22 datasets were identified that showed clear inflow signals in at least one of the bottom two slices (Figure 1). These 22 datasets were derived from 17 unique patients (12 males, 5 females, age 64.6 ± 8.3 years), 14 of which were acquired prior to radiotherapy and eight postradiotherapy (Table 1).

**FIGURE 3:**
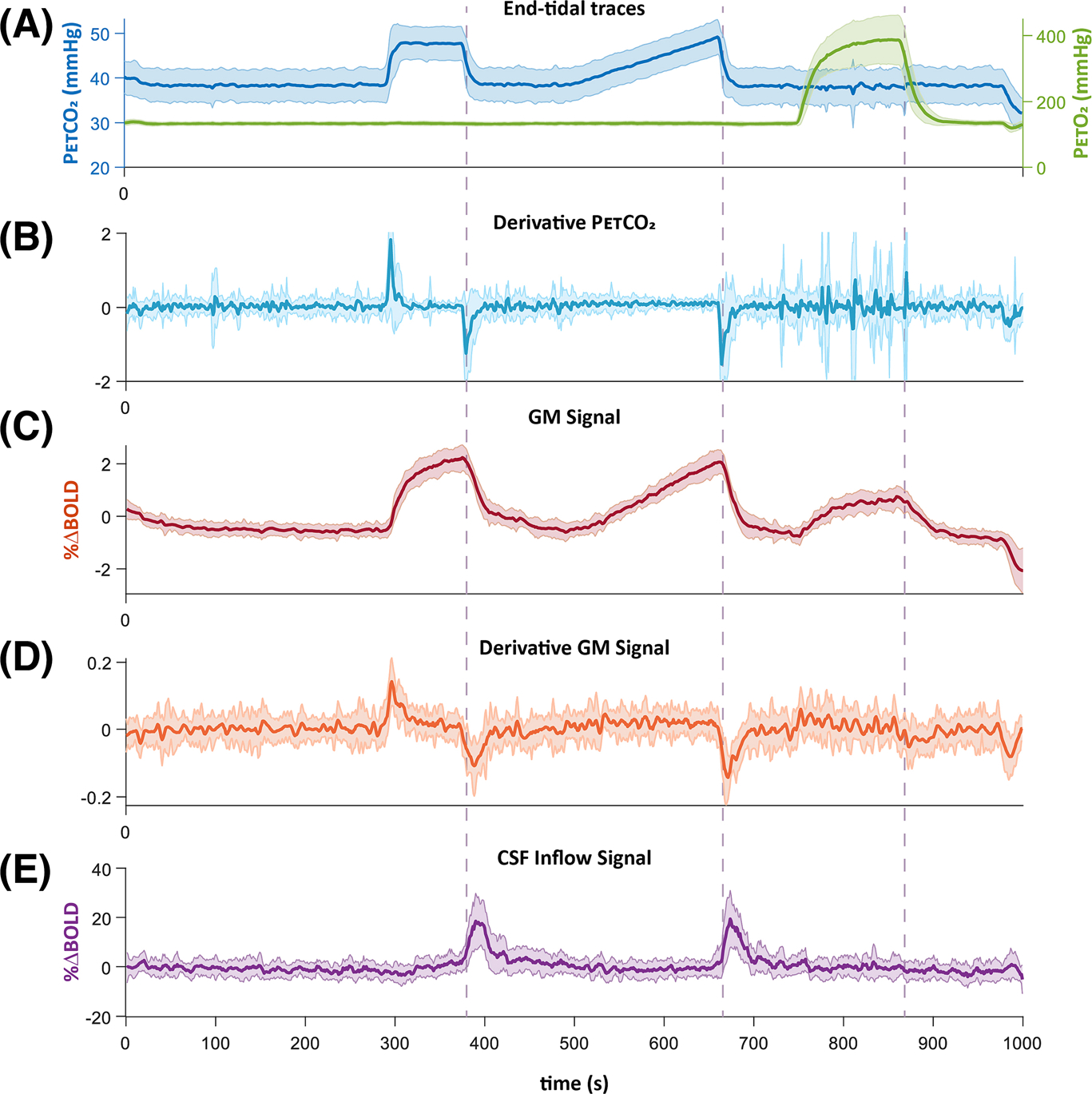
Average responses of 17 patients: (A) Group-averaged end-tidal CO_2_ (blue) and O_2_ (green) traces, and (B) The corresponding time derivative of the CO_2_ trace. (C) Group-averaged gray matter blood oxygen level-dependent (GM BOLD) signal response, and (D) The corresponding time derivative. (E) The inflow signal resulting from CO_2_-mediated modulation of cerebral blood volume (CBV). Note the timings between the peak response time-derivative (B and D) and peak inflow signal (E) and the fact that transient O_2_ produces no inflow effect. Outflow effects are absent as changes in the cerebrospinal fluid (CSF) signal are based on the time-of-flight effect, requiring the inflow of fresh, nonsaturated spins. The shaded areas indicate the standard deviation across subjects. The vertical dashed lines indicate the end of the hypercapnic and hyperoxic block.

Variations in PetCO_2_ levels resulted in CSF inflow measured near the fourth ventricle. Notably, the timing of the two CSF inflow peaks aligned with both the negative change in CBV (as indicated by the temporal BOLD signal derivative) and in PetCO_2_. Variations in PetO_2_ levels, however, did not result in measurable CSF inflow signal (Figure 3). The CSF inflow signal was delayed by 1.26 ± 1.89 s with respect to the maximum BOLD signal derivative (Figure 3D,E), with a mean correlation of 0.39 ± 0.11. The delay between the CSF inflow peak and the peak of the PetCO_2_ derivative was 5.81 ± 3.37 s (Figure 3B,E), with a mean correlation of 0.24 ± 0.08.

**FIGURE 4:**
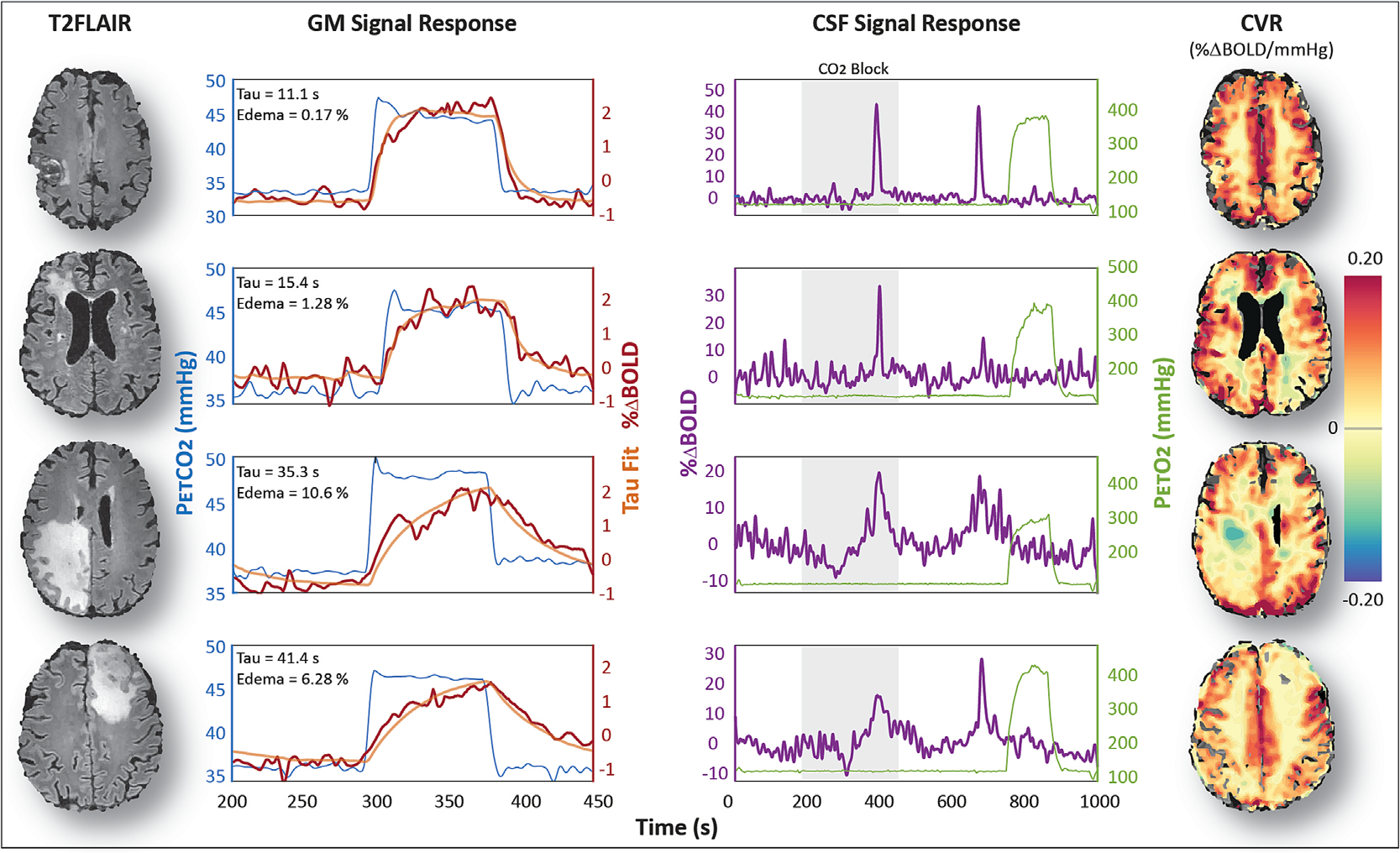
Edema and cerebrovascular reactivity (CVR) in four patients: transversal T2FLAIR slice of four patients having different edema loads. The end-tidal CO_2_ trace (blue), together with the blood oxygen level-dependent (BOLD) response (red) and the corresponding exponential fit (orange) for the hypercapnic block between imaging volume 200–450, are shown for each subject. Note that tau increases with the percentage of edema (see the value reported in the figure inset). The PetO_2_ trace (green) and the corresponding cerebrospinal fluid (CSF) inflow signal (purple) are shown for the entire protocol. Note the lack of strong inflow peaks during the hyperoxic period. The CVR maps, shown on the right, were calculated using seeVR toolbox^20^ of the corresponding slices to highlight the spatial variability in BOLD response. CVR is decreased and CSF inflow peaks become less sharp for higher edema loads. Moreover, the presence of negative BOLD responses in patients with considerable amounts of edema indicates mixed changes in cerebral blood volume caused by the presence of vascular steal. This may explain the increases in the width of the CSF response peak.

**FIGURE 5:**
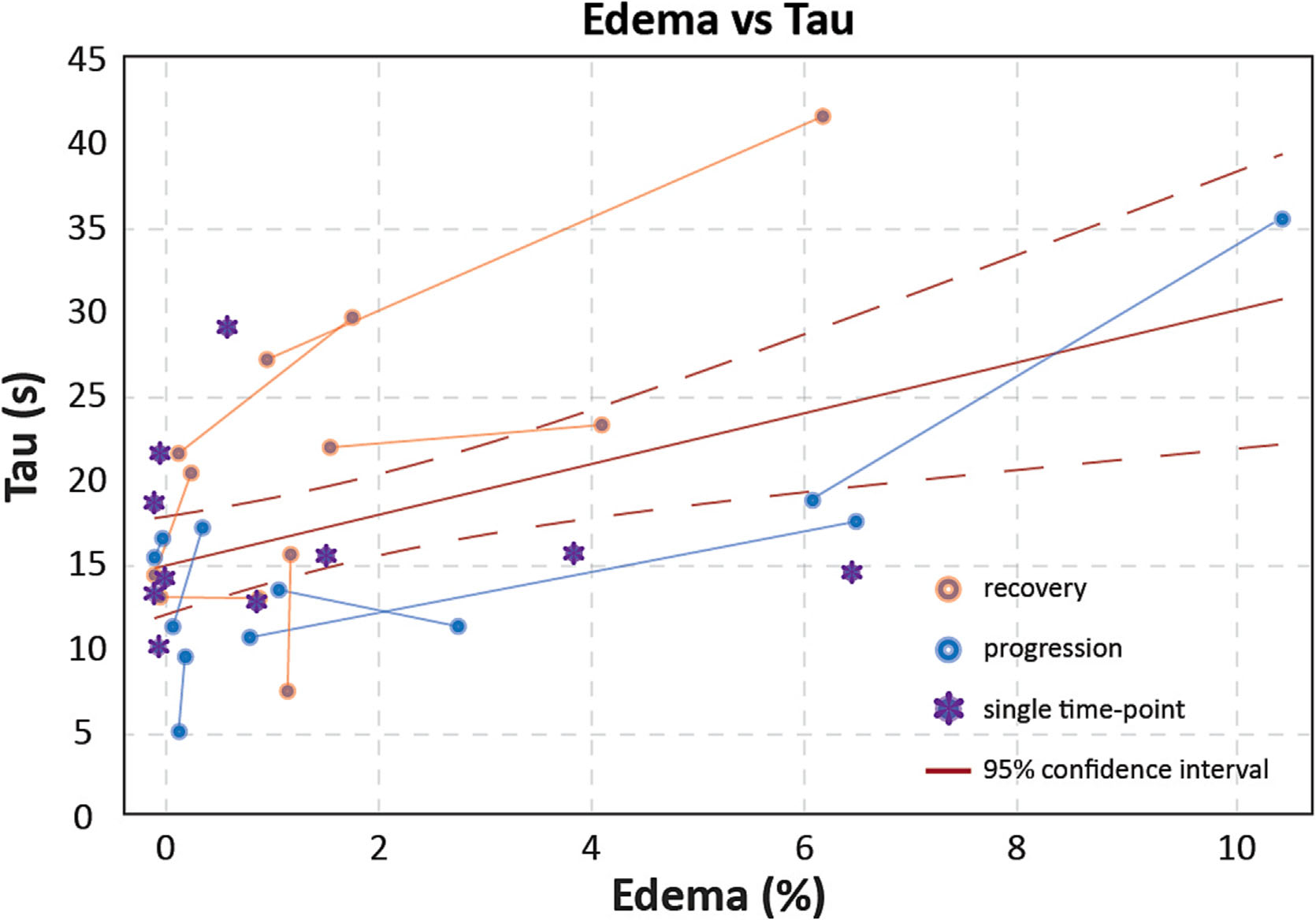
Edema versus tau for all patients: lines indicate the two time points (prior and postradiotherapy) belonging to the same patient. Orange lines indicate a decrease in edema load and blue an increase. Single time point subjects are shown in purple. *R*^2^ = 0.25, *p* = 0.003.

To assess the effect of changes in tissue properties resulting from the formation of edema, vascular dynamics were compared with edema load and CSF inflow dynamics. In cases where T2FLAIR images showed noticeable regions of edematous tissue, the vascular response, indicated by the time constant tau, was lower. Concurrently, these patients also showed affected CSF inflow dynamics in terms of a broader and lower amplitude CSF inflow peak. Moreover, regions of edema showed reduced CVR (Figure 4). When evaluated for all datasets, the vascular response was significantly slower (*R* ^2^ = 0.25, *p* = 0.003) in cases of higher edema load (Figure 5). In 10 out of 12 patients, this positive correlation was also observed between the predataset and postdataset, where treatment-related recovery was significantly correlated to a decrease in tau and progression to an increase (paired *t*-test: *p* = 0.002). In subjects where CSF inflow signal was found, the HWHM of the CSF inflow peak was not significantly correlated to the vascular dynamics, as measured by tau (*R* ^2^ = 0.08, *p* = 0.21; see also Data S1).

## 4 | DISCUSSION

The main goal of this study was to examine the relationship between vascular response and CSF flow using arterial gas challenges during BOLD MRI. This study is the first to explicitly demonstrate that modifying CBV by manipulating PaCO_2_ leads to CSF motion near the fourth ventricle, establishing a direct link between vascular responses and CSF flow. The secondary objective was to explore the relationship between brain hemodynamics, CSF flow, and changes in brain composition caused by the development of peritumoral edema. We observed a significant correlation between edema load and the speed of the hemodynamic response to alterations in PaCO_2_. These findings shed new light on the interplay between CSF and hemodynamics and edema formation, expanding the understanding of cerebral physiology.

Our findings provide further support for previous studies demonstrating a correlation between changes in hemodynamics and CSF flow.^12,13,21,22^ Prior research suggested that the (low frequency) oscillations observed in CSF flow are linked to cerebrovascular activity, possibly driven by variations in PaCO_2_ levels.^12,13^ The positioning of the BOLD imaging slab in the experiments made them sensitive to CSF inflow (rather than outflow). Specifically, reductions in arterial CO_2_ towards baseline levels triggered a vasoconstrictive response, leading to a decrease in CBV. The observed inflow signal provided explicit evidence confirming the association between PaCO_2_ and neurofluid flow, aligning with the model and measurements proposed by Yang et al.^21^ The observed delay between negative changes in BOLD signal and CSF inflow was consistent with previous findings in both awake and sleeping states.^8,21,22^

In addition to hypercapnic stimuli, we also applied controlled increases in PaO_2_ levels. While prolonged hyperoxic stimuli can lead to vasoconstriction of cerebral blood vessels, short periods of elevated O_2_ are not considered vasoactive. During hyperoxia, excess O_2_ is dissolved in the blood plasma, which can be taken up to meet the metabolic demand of tissues. This occurs at the expense of hemoglobin-bound O_2_, leading to higher venous hemoglobin saturation and a concurrent increase in the BOLD signal. Importantly, this BOLD signal change is driven by direct saturation changes, rather than by changes in the oxygen extraction fraction caused by increased CBV or CBF, as observed when applying hypercapnic states or during functional experiments. The absence of appreciable inflow effects in response to the hyperoxic stimulus served as validation for this distinction and allowed us to ensure that the inflow signals were not confounded by partial volume effects weighted by venous BOLD contributions. Therefore, while hypercapnic states demonstrated direct effects on CBV and CBF, the hyperoxic stimulus helped to confirm the specificity of our observations.

In addition to establishing a direct link between CO_2_-mediated vascular responses and CSF flow, we have uncovered new evidence suggesting that changes in tissue properties, resulting from the formation of edema, affect both the cerebrovascular response and CSF dynamics. To investigate this, we leveraged the varying edema loads in the patients within our cohort. Analyzing the CVR response dynamics, we observed that individuals with higher edema loads exhibited a slower BOLD response (represented by the time constant, tau) to changes in PaCO_2_, in contrast to those with minimal or no edema. An explanation for this positive correlation may involve compensatory mechanisms in response to increased fluid volume from peritumoral edema. It is plausible that a reduction in other fluid compartments, such as blood or CSF/ISF, or a combination of both, occurs.^24^ Although in this case the intracranial volume will be the same, the brain compliance will decrease, making it more difficult to quickly accommodate transient volume changes from CBV dynamics. It is also important to note that patients with a higher tumor and edema load demonstrated reduced baseline perfusion (see previous research focusing on a subset of the same patient cohort^16,25^) and negative BOLD responses in associated regions (Figure 4). This effect can arise because of flow redistribution, also known as “vascular steal”, which occurs in regions where the CVR response is impaired.^26,27^ While it is challenging to show that negative BOLD signals are associated with a reduction in CBV (instead of only CBF), the negative BOLD signals are at least consistent with the notion of an impaired ability of the vasculature to regulate blood flow. Finally, not all patients with high edema load represented a noticeable reduction in the rate of the CVR response. This may have been because our analysis primarily centered on presumed healthy GM tissue and did not consider potential confounding effects related to age, gender, lifestyle (e.g., smoking), or previous radiation exposure, all of which are factors that may explain some intersubject variability. Nonetheless, in patients who underwent repeated measurements, changes in tau showed a significant positive correlation with changes in edema load, lending evidence to the notion that CSF flow, vascular dynamics, and brain tissue properties are intricately linked to form a co-dependent system. Consequently, any pathological disorder that affects the cerebral vasculature, such as cerebral amyloid angiopathy or small vessel disease, may then also affect CSF flow, and possibly clearance, assuming that CSF flow is a major driver of this phenomenon. Similarly, mechanisms affecting CSF flow may then also propagate to induce vascular impairment.

Our observations reveal a correlation between changes in CBV and CSF flow, and support the notion that widespread changes in the brain’s mechanical properties may influence the vascular response. Specifically, either the formation of edema could reduce intracranial compliance, subsequently affecting both CVR and CSF flow, or restricted neurofluid flow may create increased resistance against dilation or contraction and thereby influence the vascular response. Conversely, restricted fluid flow could contribute to the formation of edema, thereby creating a negative feedback loop. It is important to emphasize that these are speculative notions, and the exact underlying sequence of events remains unclear. Nevertheless, these findings highlight the potential for incorporating brain mechanical properties, CSF flow, and cerebrovascular responses as part of an integrated system, through which to understand disease processes.

### 4.1 | Limitations

We have shown the relationship between CVR and inflow (Figure 3) and the relationship between CVR dynamics and brain tissue properties (Figures 4 and 5), and have attempted to draw a relationship between CVR dynamics and CSF dynamics by comparing the tau parameter with the width of the inflow peak. For the latter, we did not observe a significant relationship, which may reflect limits in the sensitivity of our measurement to artifacts. This is a risk in studies employing hypercapnic challenges, because exposure to elevated CO_2_ can lead to hyperventilation and, therefore, a greater chance of motion and partial volume effects. Given the 17 subjects in which CSF inflow data were available, we were probably simply underpowered for this analysis. This was probably because our data were affected by inconsistent planning between patients, and that the slices were not always exactly perpendicular to the fourth ventricle; indeed, most of the inflow effect was measured in the central canal below the fourth ventricle. Moreover, our 1050-ms TR is suboptimal for capturing the CSF dynamics because of a lack of temporal resolution. For this, shorter TRs (~400 ms) have typically been used.^12,21,22,28^ We recommend future studies with larger datasets and more optimal protocols to investigate the CSF flow dynamics in the context of the temporal characteristics of the CVR response.

It is crucial to acknowledge that the total brain metastases volume was not factored into our analysis because post-treatment CT imaging, upon which tumor volumes are delineated, is not part of the standard clinical protocol at our center. Additionally, the effect of radiotherapy on healthy vasculature is unknown. In a previous study focusing on a subset of the same patient cohort, no significant change in CVR was found between preradiotherapy and postradiotherapy.^16^ Consequently, the focus of the study was primarily on changes related to edema. However, changes in total tumor mass will also influence mechanical properties and could have implications for CVR and CSF flow. To gain a more comprehensive understanding, future studies should obtain both preradiotherapy and postradiotherapy tumor segmentations to differentiate the parallel effects of edema and tumor volume.

## 5 | CONCLUSION

Our findings reveal an association between CSF flow at the fourth ventricle and CO_2_-mediated changes in CBV that is consistent with the principles of the Monro–Kellie doctrine. This indicates that arterial CO_2_ oscillations are an important driver of CSF flow. Conversely, variations in O_2_ levels did not exhibit any discernible effect on CSF inflow, suggesting that brief periods of hyperoxia do not significantly affect CBV. To date, research related to clearance has predominantly focused on understanding its pathways and motive forces. However, the relationship between changes in tissue properties, vascular dynamics, and CSF flow has been overlooked. We found that patients presenting with higher levels of edema had slower hemodynamic responses as assessed through CVR measurements. Because we confirm that vasoactivity is a driver of CSF flow, and CSF flow is assumed to be an underpinning mechanism of brain clearance,^29^ we conclude that changes in vascular or brain mechanical properties may have implications for brain clearance in our studied patients. Moreover, these findings may also extend to individuals’ cerebrovascular or neurodegenerative diseases, in which tissue mechanics and/or vascular function are altered.

## Supplementary Material

supplementary

## Figures and Tables

**TABLE 1:** Data inclusion.

	Predatasets	Postdatasets	Total datasets	Unique patients

CSF inflow (*N*)	14	8	22 (Figure 3)	17
Vascular response (*N*)	22	12	34 (Figure 5)	22

*Note:* The number of datasets used for each analysis (CSF inflow and vascular response) coming from either preradiotherapy or postradiotherapy scans, together resulting in the total number of datasets. The number of unique patients included for each analysis is reported as well. Datasets that did not show inflow signal were excluded from the inflow analysis.

Abbreviation: CSF, cerebrospinal fluid.

## Abbreviations:

BOLD: blood oxygen level-dependent
CBF: cerebral blood flow
CBV: cerebral blood volume
CSF: cerebrospinal fluid
CVR: cerebrovascular reactivity
ISF: interstitial fluid
PaCO_2_/PaO_2_: partial pressure of carbon dioxide/oxygen
PetCO_2_/PetO_2_: end-tidal pressure of carbon dioxide/oxygen
