# Supplementary material for: CO2 as an engine for neurofluid flow: Exploring the coupling between vascular reactivity, brain clearance, and changes in tissue properties
Source: NMR Biomed. Author manuscript; Available in PMC 2024 Aug 1. (PMC11236526; doi:10.1002/nbm.5126)
Supplement: supplementary [file NIHMS1982082-supplement-supplementary.docx]

**Supplementary Info**

**S1 – Inflow signal fit**

To parametrize the inflow signal we fit a baseline and a skewed normal distribution in Matlab using the following code:

options = optimoptions('lsqcurvefit','Display','none','FunctionTolerance',...

1.0000e-8, 'StepTolerance', 1.0000e-8, 'MaxIter',1000);

model = (@(a,t) (a(5)+t*a(6))+(a(4)*(2 * (1 / (a(2) *...

sqrt(2 * pi)) * exp(-(t - a(1)).^2 / (2 * a(2)^2))) .*...

normcdf(a(3) * (t - a(1)), 0, a(2)))));

%a(1) = mu

%a(2) = sigma

%a(3) = alpha

%a(4) = scaling parameter

%a(5) = baseline offset

%a(6) = baseline slope

%t    = time

a0 = [ 150 16 0 2*max(inflow_signal) 0 0];

lb = [ (a0(1) - 20)  0 0 max(inflow_signal) -Inf -Inf];

ub = [ (a0(1) + 20) Inf Inf Inf Inf Inf];

fitresult = lsqcurvefit(model,a0,t,inflow_signal,lb,ub,options);

**S2 – Patient baseline characteristics**

| **Patient** | **Post dataset available** | **Age (years)** | **Sex (F/M)** | **Inflow pre/post** | **Edema**  **pre/post (%)** | **Primary tumor** | **Extra cerebral metastases** |
| --- | --- | --- | --- | --- | --- | --- | --- |
| 1 | Y | 56 | F | Y/Y | 1.17/2.85 | Gynecological (cervix) | - |
| 2 | Y | 63 | F | N/Y | 0.34/0 | Lung (NSCLC – adeno) | Lymphoma, adrenal gland |
| 3 | Y | 57 | F | Y/Y | 4.20/1.65 | Lung (NSCLC – adeno) | Lymphoma |
| 4 | Y | 66 | M | Y/N | 0.90/6.60 | Melanoma | Liver, peritoneal, adrenal gland |
| 5 | Y | 81 | M | N/Y | 6.19/10.60 | Melanoma | - |
| 6 | Y | 62 | F | N/Y | 1.86/0.23 | Lung (NSCLC – non-adeno) | Lymphoma |
| 7 | Y | 72 | M | Y/Y | 6.28/1.06 | Kidney | - |
| 8 | N | 67 | M | Y | 0.10 | Lung (NSCLC – adeno) | Bone, lymphoma |
| 9 | N | 74 | M | N | 0.96 | Lung (NSCLC – adeno) | Lymphoma |
| 10 | N | 52 | F | Y | 3.94 | Lung (NSCLC – adeno) | Bone, lymphoma |
| 11 | N | 58 | M | N | 0.04 | Melanoma | Lung |
| 12 | Y | 75 | M | Y/Y | 0.17/0.45 | Melanoma | Lung, bone, liver |
| 13 | N | 61 | M | Y | 0.05 | Gastrointestinal (appendix) | - |
| 14 | N | 66 | M | Y | 0.68 | Lung (SCLC) | - |
| 15 | Y | 53 | M | Y/Y | 0/0.08 | Kidney | - |
| 16 | Y | 72 | M | Y/N | 0.98/0.05 | Lung (NSCLC – adeno) | - |
| 17 | N | 65 | M | N | 6.55 | Gastrointestinal (colon) | - |
| 18 | Y | 75 | M | Y/N | 1.28/1.25 | Lung (NSCLC – adeno) | - |
| 19 | N | 74 | F | N | 1.61 | Lung (NSCLC – adeno) | - |
| 20 | Y | 62 | M | Y/N | 0.23/0.29 | Melanoma | Lung, bone, lymphoma |
| 21 | N | 59 | M | Y | 0 | Kidney | Lymphoma, adrenal gland, soft tissue |
| 22 | N | 76 | F | N | 0 | Melanoma | - |

**
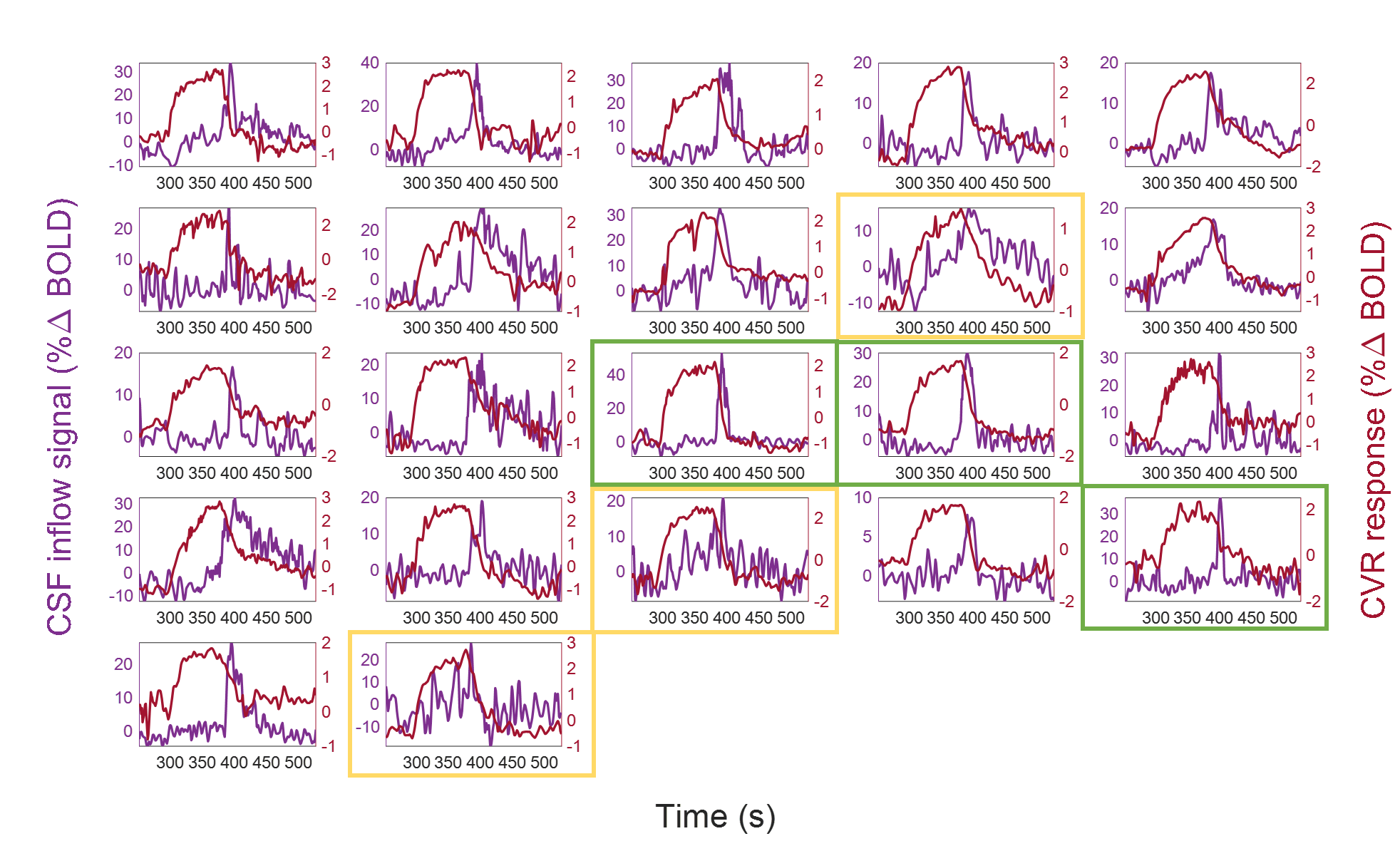
**

**S3 – CSF inflow peaks** **for all 22 datasets**: De-noised GM BOLD signal and CSF inflow signal in the protocol range of imaging volume 250 – 525. While some subjects show clear and distinctive inflow peaks with steady baselines (highlighted in green), others show broader and more irregular peaks (highlighted in yellow). Notably, these peaks start before the end of the BOLD response. This is unlikely because it suggests that CSF will enter the brain before blood has exited, leading to an increase in cerebral volume which is not in line with the Monro-Kellie doctrine. These peaks might be caused by motion we noticed in our data and could explain why a significant relation was not found between the width of the CSF peak and tau.
